# Three dimensional visualization of subcellular and suborganellar structures in near-native plant cells by synchrotron soft X-ray tomography

**DOI:** 10.3389/fpls.2025.1705667

**Published:** 2025-12-01

**Authors:** Mo Da-Sang Hua, Yi-Hung Lin, Chia-Chun Hsieh, Chang-Lin Chen, Zi-Jing Lin

**Affiliations:** X-ray Imaging Group, Experimental Facility Division, National Synchrotron Radiation Research Center, Hsinchu, Taiwan

**Keywords:** soft X-ray tomography, protoplast, plant cell, near-native cells, ultrastructure

## Abstract

Significant advances in photosynthesis research have been achieved using reverse genetics alongside cellular imaging techniques such as confocal microscopy and transmission or scanning electron microscopy. However, these conventional imaging methods often require chemical fixation or physical sectioning, which can alter the native endocellular ultrastructure and limit the ability to observe subcellular environments. In this study, we utilized synchrotron-based soft X-ray tomography (SXT) at the TPS-24A1 beamline of the National Synchrotron Radiation Research Center (NSRRC), Taiwan, to non-destructively visualize three-dimensional ultrastructure of protoplasts isolated from *Arabidopsis thaliana* and *Bidens pilosa* in their near-native state. Our results revealed marked differences in organelle morphology and spatial arrangement compared to traditional electron micrographs and confocal images, highlighting the robustness and volumetric resolution of SXT for near-native structural analysis. Importantly, this method enabled visualization of organelles and sub-organelle compartments such as chloroplast thylakoid systems and lipid bodies without chemical fixation or staining. The discovery of previously unreported large vesicular structures in *B. pilosa* chloroplasts further demonstrates the power of SXT in uncovering novel species-specific ultrastructural features. Overall, this combined approach of protoplast isolation and cryogenic SXT offers a powerful tool for advancing our understanding of photosynthetic machinery and cellular organization. Moreover, it opens new opportunities for studying plant-microorganism interactions, metal uptake, and intracellular biochemical dynamics with minimal sample perturbation.

## 1 Introduction

Over the past decade, plant biologists have increasingly focused on the subcellular interactome of photosynthetic organelles using advanced imaging techniques such as confocal microscopy and transmission electron microscopy (TEM). Thylakoids, the fundamental units of oxygenic photosynthesis across phototrophs, have been extensively characterized via TEM to elucidate their structural components (Bussi et al., 2019). In higher plants, chloroplast thylakoids are organized into tightly stacked grana, which distinguish them from their prokaryotic counterparts (Patty et al., 2019; Koochak et al., 2019). TEM observations reveal grana stacks as bellow-like structures, interconnected by unstacked stroma lamellae forming right-handed helices (Austin and Staehelin, 2011; Bussi et al., 2019). The lumen, a narrow aqueous inner space lining each granum and separated by stromal fluid-filled partition gaps, may be held together by van der Waals forces, although further evidence is required (Nevo et al., 2012; Puthiyaveetil et al., 2017).

Within chloroplasts, multiple sheets of stroma lamellae connect grana stacks through narrow slits known as frets, forming a continuous thylakoid membrane system and potentially facilitating luminal communication via left-handed helices (Bussi et al., 2019). While TEM and related electron microscopy techniques offer high-resolution visualization of thylakoid architecture, they necessitate extensive sample preparation, including chemical fixation, dehydration, and ultrastructural labeling (Weigel and Glazebrook, 2010; Wilson and Bacic, 2012). Investigations into plant organelle dynamics and cellular metabolism under environmental fluctuations have largely relied on these microscopic approaches (Jamil et al., 2024; Wang et al., 2023; Koenig et al., 2023; Perico and Sparkes, 2018). These tools provide spatial resolutions ranging from nanometers to micrometers, enabling structural analysis of proteins and organelles alike (Sung et al., 2015; Graham and Orenstein, 2007). More recently, cryogenic electron microscopy (cryo-EM) has emerged as a powerful alternative, enabling imaging of biological samples in a near-native, hydrated state without chemical fixation (Bai et al., 2015).

Confocal microscopy, especially when combined with fluorescently tagged proteins and dyes, has been instrumental in studying pathogen responses (Hardham, 2012), cytoskeletal organization, and molecular dynamics in plant cells (Colin et al., 2022; Wymer et al., 1999). However, it remains limited in studies of organelle dynamics during photomorphogenesis due to phototoxicity, limited availability of fluorescent probes, and spectral overlap (Cammarisano et al., 2021). Similarly, heavy metal uptake and metabolism in roots can be affected by such imaging constraints (Chao et al., 2014). Furthermore, the chemical fixation required for TEM may induce structural artifacts, making it challenging to capture dynamic interactions among organelles (David and Williams, 2009). Consequently, cryo-EM is increasingly favored for elucidating ultrastructure in a near-native state, particularly for large protein complexes isolated via vitrification rather than chemical fixation (Wang et al., 2025).

An advanced extension of cryo-EM, cryogenic electron tomography (cryo-ET), now allows three-dimensional visualization of large subcellular structures with improved resolution (Villa et al., 2013; Rubinstein, 2017; Sanchez Carrillo et al., 2023). Recent application of cryo-ET to *A.thaliana* root protoplasts revealed detailed native organelle and sub-organelle structures (Sanchez Carrillo et al., 2023). Nevertheless, cryo-EM remains limited by sample thickness constraints—typically 50–100 nm—leading to a narrow field of view along the z-axis, which hampers volumetric imaging of entire plant cells. To address this challenge, synchrotron-based soft X-ray tomography (SXT) has emerged as a powerful, non-invasive imaging modality (Larabell and Nugent, 2010; Loconte et al., 2021, 2022; Weinhardt and Larabell, 2025). SXT bridges the gap between electron and light microscopy, enabling three-dimensional visualization of subcellular organization in hydrated, chemically unaltered samples. It requires no staining, fluorescent tags, or fixatives, thus preserving a near-native cellular architecture. Additionally, SXT circumvents the limited penetration depth of cryo-ET and provides a z-axis resolution suitable for observing large eukaryotic cells. Recent developments in high-angular soft X-ray projection tomography now enable imaging of biological samples up to 10 µm thick, generating high-contrast three-dimensional reconstructions. However, plant cells—often ranging from 10 to 100 µm in size—remain challenging due to their substantial thickness (Sakai et al., 2019; Wu et al., 2009). While TEM requires ultra-thin sectioning of tissue (Zechmann, 2023) and cryo-ET relies on protoplast isolation (Sanchez Carrillo et al., 2023), SXT holds promise if suitable cellular dimensions can be achieved.

In addition to SXT, two other advanced three-dimensional imaging modalities—focused ion beam–scanning electron microscopy (FIB-SEM) and serial block-face SEM (SBF-SEM)—have been developed for volumetric ultrastructural analysis. Both techniques employ sequential surface removal and imaging, allowing nanometer-scale 3D reconstruction of biological samples. FIB-SEM achieves extremely high spatial resolution (3–10 nm) through precise ion-beam milling, ideal for resolving sub-organellar details but limited to relatively small volumes and requiring destructive sample processing. SBF-SEM, in contrast, utilizes automated diamond-knife sectioning to image larger volumes (tens to hundreds of micrometers) at slightly lower resolution (10–30 nm), making it particularly suitable for tissue-level or multicellular reconstructions. Compared with these resin-embedded, heavy-metal-stained methods, SXT offers several clear advantages: it is fully non-destructive, requires no staining or sectioning, and preserves hydrated samples in their near-native cryogenic state. Moreover, SXT uniquely provides intrinsic biochemical contrast based on differential X-ray absorption, enabling label-free visualization of organelles within intact cells. Thus, while FIB-SEM and SBF-SEM are powerful for high-resolution volumetric analysis of fixed samples, SXT stands out as the only technique capable of capturing three-dimensional cellular architecture in a fully preserved, native-like environment. Collectively, these complementary techniques provide a continuum of imaging capabilities that together advance our understanding of plant subcellular organization across scales, from fine ultrastructure to whole-cell architecture. In this study, we refined a mesophyll protoplast isolation protocol to yield smaller cells (10–30 µm), optimized for soft X-ray imaging using the beamline 24A1 of the Taiwan Photon Source (TPS-24A1). This refinement, coupled with the photon-energy-specific penetration of soft X-rays (Lai et al., 2023), enabled high-resolution, label-free visualization of native thylakoid networks and previously unreported subcellular structures within plant cells.

In summary, synchrotron-based SXT provides a powerful and non-invasive approach for investigating plant subcellular architecture in its near-native state, offering high-resolution three-dimensional insights into organelle organization and interactions. The present study demonstrates the feasibility and potential of SXT for visualizing near-native plant cell ultrastructure, thereby laying the foundation for its broader application in plant cell biology. This approach opens new perspectives for elucidating the dynamic subcellular interactome under varying physiological and environmental conditions.

## 2 Materials and methods

### 2.1 Part I: plant sample preparation

#### 2.1.1 Plant material and growth conditions

*A.thaliana* seeds were sanitized with bleach, rinsed three times with MilliQ water, and sown on an antiseptic agarose tissue culture plate containing ½ Murashige and Skoog (MS) basal salts (Murashige and Skoog, 1962), then incubated in the dark for 48 hours until seedlings developed. The seedlings of *A.thaliana* were sown in a potting mixture of vermiculite, perlite, and peat moss (1:1:2 by volume ratio) that had been wetted with distilled water. The seeds and pots were covered with cellophane wrap and stored in a dark, environmentally controlled cabinet at 22°C for 16 hours. After 16 hours, the seeds and pots were transferred to an environmentally controlled lighting chamber set to a long photoperiod (16 h light/8 h dark) at 22°C with optimal lighting (~150 µE·m^−2^·s^−1^). Fourteen-day-old *A.thaliana* plants grown under those conditions were selected for protoplast isolation. *B.pilosa* seeds were sanitized with bleach, rinsed three times with MilliQ water, and one seed was sown per pot containing a soil mixture of vermiculite, perlite, and peat moss (1:1:2 by volume ratio) wetted with distilled water. *B.pilosa* seeds were germinated in a dark cabinet for 48 hours before being moved outdoors to ensure sufficient sunlight. Fifteen-day-old *B.pilosa* plants grown outdoors, as they are required identical environmental condition as the parental plants that were naturally grown in the wild, were selected for protoplast isolation.

#### 2.1.2 Protoplast isolation

Fifteen to twenty young leaves (approximately 0.7 cm × 3 cm) from *A.thaliana* and *B.pilosa* were collected using a clean pair of scissors and placed onto a clean Petri dish. *B.pilosa* leaves were cleaned with bleach and rinsed three times with Milli-Q water in the Petri dish, since these plants are grown outdoors. Sticky tape was applied to both sides of the leaves from each species and peeled off in opposite directions to expose the mesophyll layer between the cuticle and epidermis. The taped leaf pieces were transferred into a new, labeled, sterilized Petri dish, and 10 mL of enzyme solution (1% cellulose “Onozuka” R10, 0.25% macerozyme “Onozuka” R10, 0.4 M mannitol, 10 mM CaCl_2_, 20 mM KCl, 0.1% BSA, 20 mM MES pH 5.7) was added. The Petri dish was gently shaken on a platform shaker at 40–55 rpm under light for 60 minutes until the liquid turned from creamy white to green. The Petri dish was tilted at a 15° angle, and the liquid portion was collected with a dropper and transferred into two separate 15 mL centrifuge tubes labeled by plant species. The mixture was centrifuged at 100 RCF for 3 minutes; any floating debris was removed with a dropper and discarded appropriately. The supernatant was discarded, and pre-chilled (4 °C) W5 buffer (0.9% w/v NaCl, 1.84% w/v CaCl_2_, 0.08% w/v KCl, 0.1% w/v glucose, 2 milliMolar (mM) MES; pH 5.7) was used to wash and replace the enzyme solution by gently added to the pellet. The tube was gently inverted three times to resuspend the protoplasts. Centrifuging and washing off the enzyme with W5 buffer were repeated twice more to remove residual enzymes, with the supernatant discarded after each wash. After the third centrifugation, 10 mL of W5 buffer was added to each pellet, and the green suspension was incubated on ice for 30 minutes. During incubation, unfiltered protoplasts from both species were counted with a hemocytometer under a light microscope, and the counts were recorded as to obtain the maximum yield of cells with diameter between 10 – 20 µm for soft X-ray beaming. Following incubation, each protoplast suspension was filtered through a sterilized 200 μm cell strainer (NC0776417, Fisher Scientific^®^) using a sterile syringe or dropper; note that filtration may take 2–3 hours depending on protoplast density. For *B.pilosa* 50 μm cell strainer (NC1625104) was used instead of the 200 μm, and the 20 μm filtration in the following step was skipped for *B.pilosa*. Each filtrate was centrifuged at 100 RCF for 3 minutes to concentrate the protoplasts. The supernatant was removed, and each pellet was resuspended in 10 mL of pre-chilled W5 buffer. Each suspension was filtered through a sterilized 20 μm cell strainer (NC1004201, Fisher Scientific^®^), incubated on ice, and the cells were recounted via hemocytometer; if losses exceeded 40% (maximum yield of cells with diameter between 10 – 20 µm is the goal), the procedure was restarted from after enzyme digestion. The protoplasts were then centrifuged once more and resuspended in modified MMg solution (0.4 M mannitol, 15 mM MgCl_2_, 4 mM MES, pH 5.7) to a final concentration of 2–5 × 10^5^ cells/mL. A 300 µL aliquot of the protoplast suspension was transferred into a 30 mm Petri dish containing a glow-discharged gold–holey carbon plate, and the setup was incubated overnight in a lighting chamber under a long photoperiod.

#### 2.1.3 Glow discharge of EM-grid with holey carbon film

The gold EM-grid with holey carbon film was removed from its casing and inspected for lint or bending. The grid’s orientation was turned upright (carbon film or darker non-reflective surface on top) before it is placed onto the holder for glow discharge. The equipment was powered on, and the parameters set to a current of ~15 mA, a duration of 30 seconds (with an optional 15-second hold or pre-run to stabilize the chamber), and a vacuum of 0.39 mbar. The grid was then loaded into the chamber and glow discharge was initiated, with the process via the plasma’s ionizing cloud formation once the target vacuum is reached.

#### 2.1.4 Cryogenic fixation of bio sample

*A.thaliana* and *B.pilosa* protoplasts were incubated in W5 buffer overnight under sufficient light and were cryofixed using the Leica^®^ EM GP2 automatic plunge freezer. The equipment was pre-chilled by having the small Dewar filled with liquid nitrogen, the blotting paper renewed, liquid ethane added to the cryofixation dish, and the humidity chamber set to 85% at 20°C. The gold EM-grid was clipped onto the micro-tip tweezer and then clamped onto the machine at the tweezer holder area. The “Load sample” button was pressed, and 2.0 μL of W5 buffer containing the protoplasts from each plant along with 1× 100 nm nano gold colloidal particles was added to both sides of the gold plate. The “Blot” button was pressed to blot and freeze the sample in liquid ethane, thereby producing two replicated EM-grids per plant species and a total of four EM-grids. The sample was now considered ready for SXT; however, it may be stored in a cryogenic storage unit for later viewing or pre-screened with a cryo-stage from Linkam^®^ and a light or fluorescent microscope to have protoplasts identified for soft X−ray observation.

### 2.2 Part II: soft X-ray tomography

#### 2.2.1 Post-screening

To ensure isolated protoplasts from both *A.thaliana* and *B.pilosa*, were viable before uploading to the SXT main chamber for beaming, the EM-grids containing the samples were viewed under a wide-field fluorescent microscope using a cryo-EM stage. Each grid from individual plant species was checked for a total of 10 viable cells with no defective chloroplasts according to previous study’s description: clumping and non-autofluorescence (Lung et al., 2015), and then the position of these cells were noted down so during beaming, the sample holder could be moved to the specific position on each of the EM-grid.

#### 2.2.2 Transferring the samples in the SXT microscope

The selected sample was carefully removed from the stored grid box and placed into the sample holder in liquid nitrogen for the microscope. The sample holder was transferred to the SXT vacuum chamber using the custom-made cryo-transfer device (load-lock system). The cryo-sample was transferred onto the sample base with precision and care using the microscope-equipped robotic gripper, and the optics and the sample were moved into the soft X-ray beam path using the vacuum-compatible motorized stage.

#### 2.2.3 Tomographic data acquisition

The optical microscope (OM) image was used to help locate the target cell, and the field of view (FOV) for imaging was selected. Before imaging, the motorized stages were used to align the FOV in the center of rotation, and the rotation center was positioned in the center of the CCD camera. The exposure time was set between 1 to 3 seconds based on the sample thickness and beam flux, and the acquisition parameters were set with designated start and end tilt angles, acquiring one image per degree. After data acquisition, the sample was translated to a blank region, and sets of flat-field images were captured for background correction. The acquired tilt series was processed and converted into a single stack file (*.st format) that was compatible with IMOD for tomographic reconstruction. A 2D micrograph was take from three cells on each EM-grid before the tilt angle projection images were taken, from there one of the three cells that has the best X-ray contrast projection image was selected for tilt angle projection image acquisition. In future studies however, if an in-depth study of changes in organelles or ultrastructure were to be done, between 5 to 10 cells should be observed per EM-grid for 2D micrograph; and the, between 3 to 5 cells per EM-grid should be used for tilt angle projection image acquisition.

#### 2.2.4 Material/equipment

##### 2.2.4.1 Equipment

1. Plunge freezer (EM GP2/Leica/Wetzlar, Germany).
2. Glow discharge cleaning system (PELCO easiGlow/Ted Pella/California, USA).
3. Wide-field fluorescence microscopy (Axio Imager A2 and Axio Scopy A1/Zeiss/Jena, Germany).
4. Cryo-correlative microscopy stage (CMS196V3/Linkam/Salfords, UK).
5. Quantifoil R2/2 circular holes on Au G200F1 finder grids (N1-C16nAuG1-01/Micro Tools GmbH/Großlöbichau, Germany).
6. The 100 nm gold colloid solution (EM.GC100/BBI Solutions/Crumlin, UK).

##### 2.2.4.2 Softwares

1. IMOD package (version 5.1.2 for 64-bit Windows with CUDA 12); freeware downloadable from: IMOD Download developed by Kremer et al. (1996).

2. Fiji (https://imagej.net/software/fiji/downloads).

## 3 Results

### 3.1 Modified protoplast preparation enabled soft X-ray tomography imaging of plant cells

To enable high-resolution soft X-ray tomography (SXT) of plant organelles in their near-native state, we implemented two essential modifications to the standard protoplast isolation protocol (Wu et al., 2009). These adjustments were critical for generating viable, small-sized protoplasts that fall within the optimal penetration depth of soft X-ray beams (~15 µm), thus overcoming a longstanding limitation in applying SXT to plant cells.

The first modification involved careful selection of source tissue. Young leaves, particularly the third true leaf from the shoot apical meristem, were chosen due to their high cellular activity, thin cell walls, and relatively homogeneous mesophyll cell populations. This tissue source was used for both the model plant *A.thaliana* and the non-model species *B.pilosa*, a eudicot known for its larger average cell size and denser leaf structure. The second critical modification consisted of a two-step filtration procedure aimed at enriching for smaller protoplasts. After enzymatic digestion, the crude suspension was initially passed through a 200 µm cell strainer to eliminate residual tissue fragments and large protoplasts. The filtrate was then diluted with protoplast suspension buffer and passed through a second 20 µm strainer. This sequential filtration markedly increased the proportion of protoplasts within the diameter under 20 µm, thus optimizing compatibility with the beam penetration and spatial resolution characteristics of the TPS-24A1 SXT beamline.

**Figure 1:**
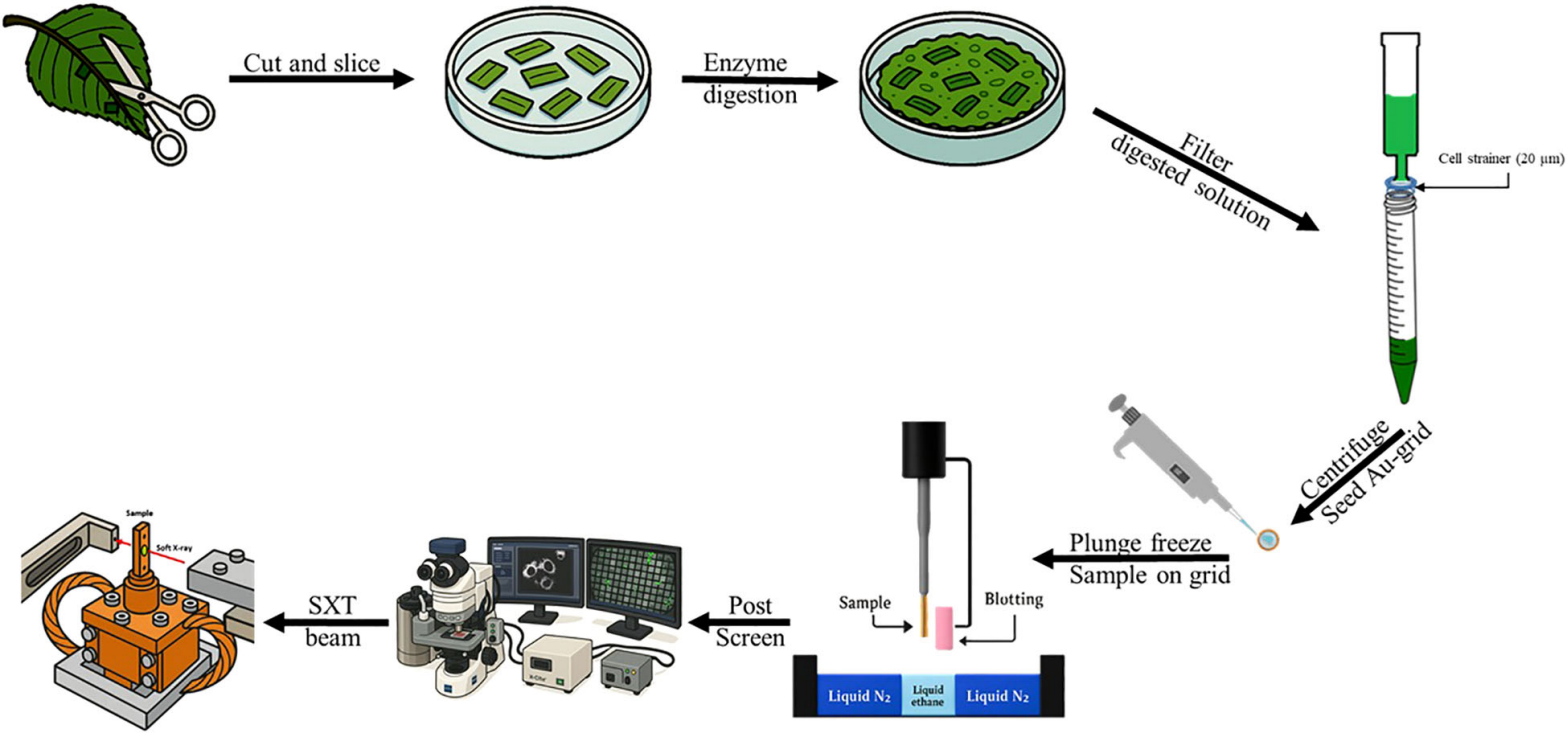
Schematic cartoon representation of the steps required for protoplast preparation to be beamed by a synchrotron radiation based soft X-ray. The third leaf from the apical meristem was collected and 3–5 leaves were used per study. The mesophyll layer was exposed via surgical scissors, scalpel and a table top Scotch ^®^ tape. Enzymatic reaction was carried out by submerging the leaf into a petri dish filled with macro enzyme and cellulase from Yakult^®^. Then the slushy mixture was decanted into a 50 mL sterile syringe and then filtered without applying pressure through a sterile cell strainer (20µm) from Falcon^®^. From there, the filtrate containing the protoplast was centrifuged and seeded unto the gold carbon plated EM grid. Later it was then plunge frozen. After which, the sample on the grid can be propped unto a Linkam ^®^ Cryo-correlative microscopic stage (Cryo-CLEM, CMS196v³) and the appropriate size protoplast can be selected for SXT. Finally, the samples on the grid was transferred into the main chamber of TPS 24A SXT beamline.

Following isolation, the filtered protoplasts were plunge-frozen in liquid ethane pre-cooled with liquid nitrogen, preserving cellular ultrastructure by inducing vitrification rather than ice crystal formation. This cryo-fixation approach avoided the use of chemical fixatives and dehydration steps that could introduce structural artifacts. Protoplasts were then screened using a wide-field cryo-fluorescence microscope to identify viable cells with no defective chloroplasts, which were subsequently positioned onto gold holey carbon EM grids for imaging. The entire workflow—from tissue selection to sample loading—was optimized to maximize preservation of near-native subcellular architecture and reduce sample loss during handling. The finalized protocol enabled direct transfer of prepared samples into the high-vacuum chamber of beamline TPS-24A1 for SXT acquisition. A detailed schematic of the stepwise procedure, including the critical modifications introduced in this study, is provided in **Figure 1**.

### 3.2 SXT captured chloroplast ultrastructure in *A. thaliana* protoplasts

**Figure 2:**
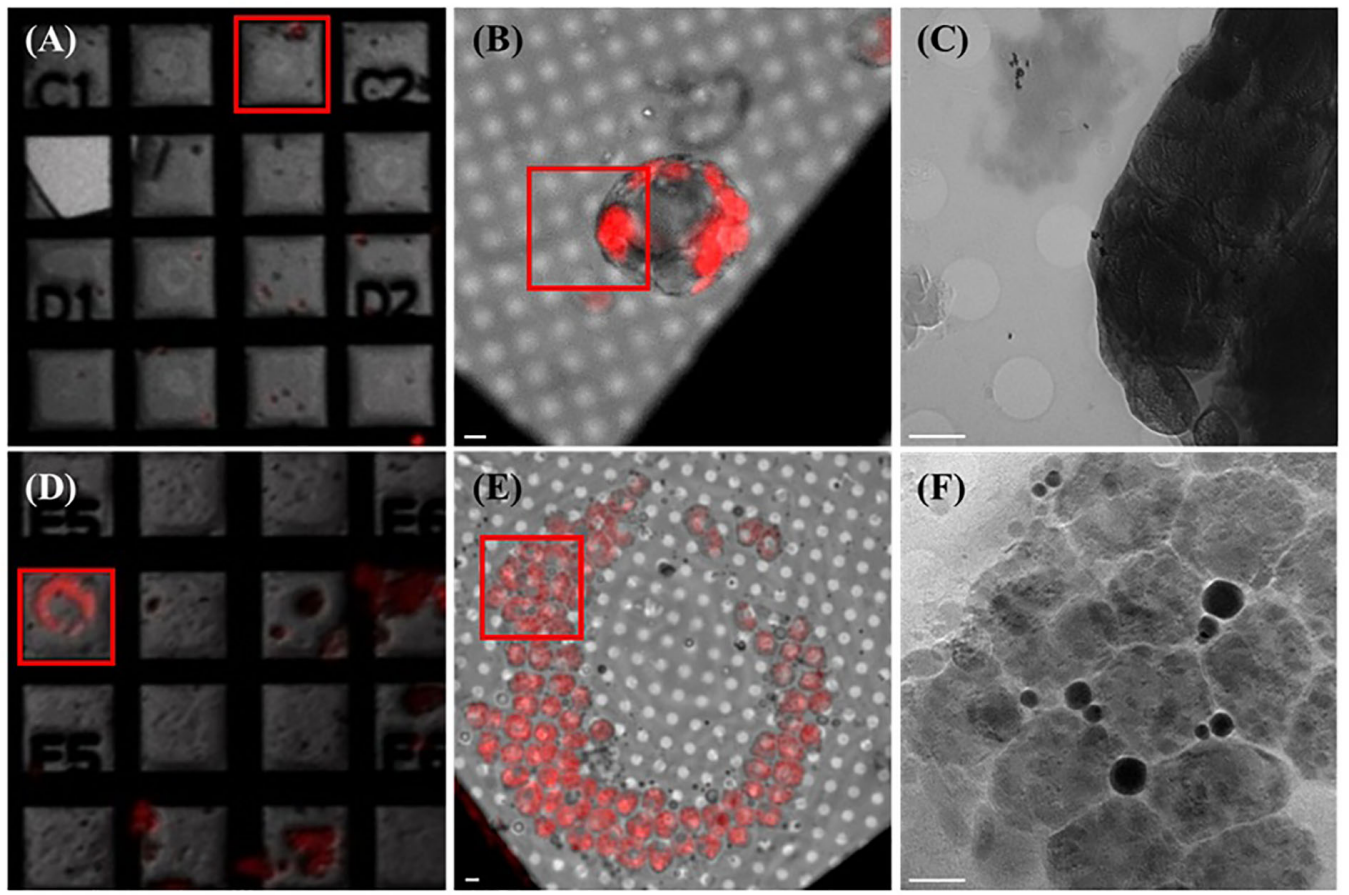
Fluorescent microscopic images of protoplasts extracted from *A.thaliana* and *B.pilosa* leaves correlate to their soft X-ray electron micrographs. **(A)** 5x magnification fluorescent microscopic image of *A.thaliana* protoplasts seeded unto the EM Au-grid. Red square mark selected mesh for 100x fluorescent microscopic image. **(B)** 100x magnification fluorescent microscopic image of *A.thaliana* protoplast seeded unto the EM Au-grid. Red square mark selected area for SXT acquisition. **(C)** Zero degree (0°)-projection electron micrograph displaying *A.thaliana*’s subcellular organelles and ultrastructure within each organelle. **(D)** 5x magnification fluorescent microscopic image of *B.pilosa*’s protoplasts seeded unto the EM Au-grid. Red square mark selected for 100x fluorescent microscopic image. **(E)** 100x magnification fluorescent microscopic image of *B.pilosa*’s protoplast seeded unto the EM Au-grid. Red square mark selected area for SXT acquisition. **(F)** Zero degree (0°)-projection electron micrograph displaying *B.pilosa*’s subcellular organelles and ultrastructure within each organelle. Scale bar for all diagrams ≈ 2 µm.

Using the natural autofluorescence of chlorophyll, *A. thaliana* protoplasts smaller than 20 µm in diameter were readily identified under 5× and 100× wide-field fluorescence microscopy (**Figures 2A, B**). These smaller protoplasts demonstrated excellent compatibility with the Au-grid substrate and were found to maintain spherical integrity after plunge freezing. Notably, 0° soft X-ray projection images from TPS-24A1 revealed distinct chloroplast (**Figure 2C**), including stroma, envelope membranes, and possible grana stacks. These features were clearly visualized in the absence of heavy-metal staining or chemical fixatives, highlighting the effectiveness of our optimized workflow for preserving near-native plant cell ultrastructure.

### 3.3 SXT visualized internal structures in larger *B. pilosa* protoplasts

To test the applicability of this protocol in non-model plant species, we extended our methodology to *B. pilosa*, which possesses considerably larger mesophyll cells. Due to their size, only a single filtration step (50 µm instead of the 200 + 20 µm) was used. Fluorescent microscopy revealed large, spherical protoplasts ranging from 25–35 µm in diameter, which were still compatible with the Au-grid but posed greater challenges in terms of full beam penetration (**Figures 2D, E**). Despite this, TPS-24A1’s high-flux soft X-ray beam was able to acquire clear projection images of *B. pilosa* protoplasts, revealing internal features not previously visualized in plant cells of this size. Notably, the projection images captured discrete vesicle-like structures and electron-dense regions within chloroplasts that were later identified as lipid bodies (**Figure 2F**). This result demonstrates the potential of SXT for studying organelle morphology even in non-model plants with large protoplasts.

### 3.4 3D tomographic reconstruction revealed detailed subcellular organization

To generate three-dimensional tomograms, tilt-series images were acquired by rotating the protoplasts within a range of −60° to +60°for A. thaliana and −65° to +65°for B. pilosa. These datasets were reconstructed using IMOD software (Kremer et al., 1996), enabling volumetric visualization of internal organelles with nanometer-scale resolution across a depth exceeding 10 µm.

**Figure 3:**
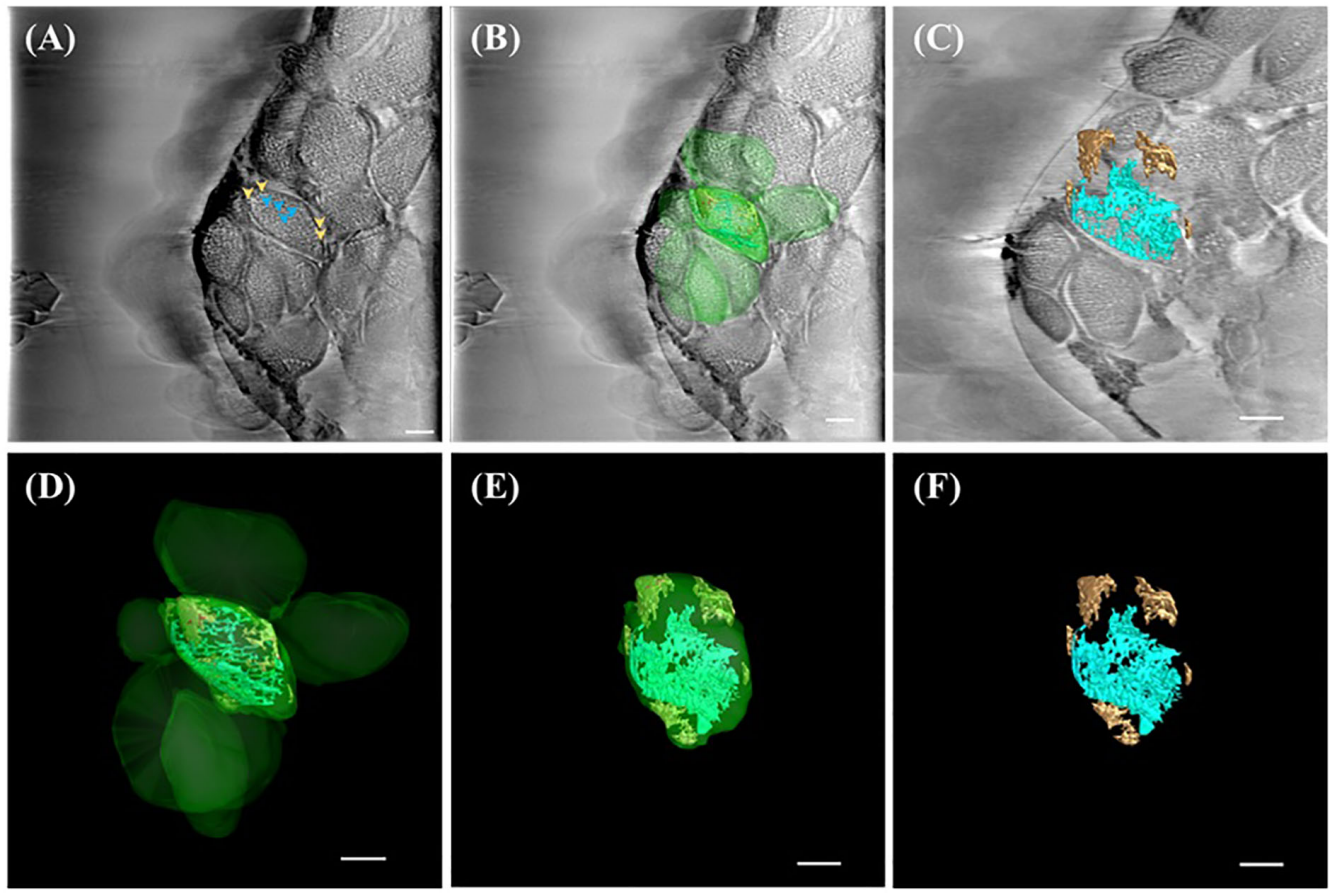
SXT showing three-dimensional (3D) representation of ultrastructure within the chloroplast of *A.thaliana*. **(A)** Reconstructed image of the 0°-projection SXT acquired from TPS24A electron as 2D micrograph (slice 125/250) showing thylakoid (cyan arrowhead) and starch granules (orange arrowhead) according to the X-ray absorbance level. **(B)** 3D representation of chloroplasts (green), thylakoid system (cyan) and starch granules (brown) overlaid on the reconstructed electron micrograph (slice 125/250). **(C)** 3D representation of the reconstructed tomography tilted at -49° off the x-axis and overlaid on slice 75/250, showing the starch granule (brown) and thylakoid system (cyan). **(D)** 3D representation of chloroplasts (green), thylakoid system (cyan), and starch granule (brown). **(E)** 3D representation of one chloroplast (green) containing thylakoid system (cyan) and starch granules (brown) tilted at -49° off the x-axis, showing the outer membrane (green), starch granule (orange) and thylakoid system (cyan). **(F)** 3D representation of the thylakoid system (cyan) and starch granules (brown) tilted at -49° off the x-axis. All scale bar in **Figure 3** = 1 µm.

In *A. thaliana* chloroplasts, reconstructed cross-sections revealed spherical starch granules (marked by orange arrowheads) surrounded by complex, network-like thylakoid membranes (gray patches, blue arrowheads) (**Figures 3A, B**). These structures were spatially segregated within the chloroplast stroma, consistent with previous electron microscopy studies but now captured without the need for chemical fixation. Subsequent segmentation and 3D rendering of the tomograms provided a comprehensive view of chloroplast organization, showing thylakoid systems (cyan) traversing the stroma in interconnected helices and starch granules (orange) clustered near the envelope membrane (**Figures 3C–F**; **Supplementary Videos S1**, **S2**). The 3D visualization also allowed identification of subdomains within the chloroplast, suggesting differential compartmentalization of metabolic functions.

### 3.5 Comparative analysis reveals species-specific ultrastructure in *B. pilosa*

**Figure 4:**
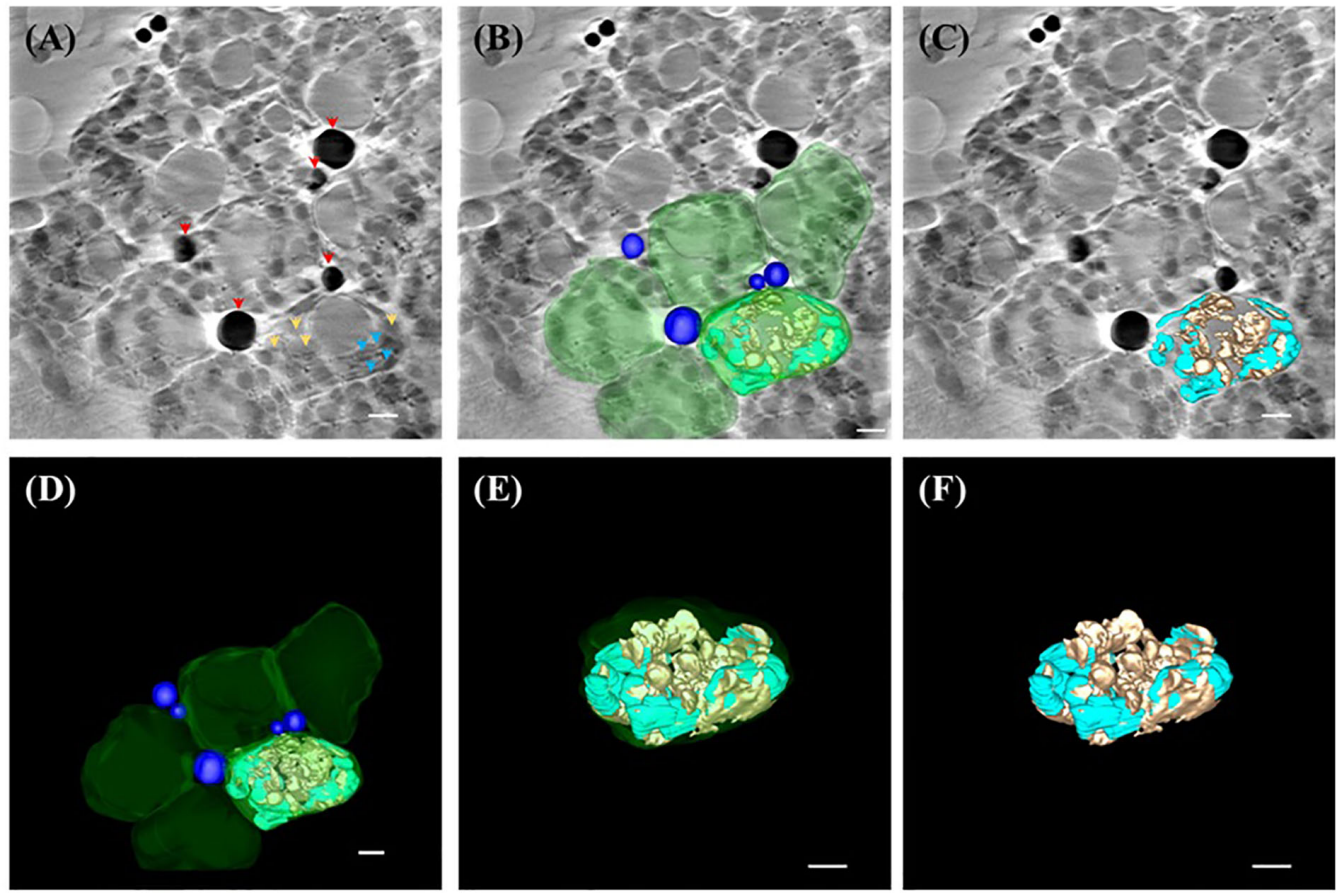
SXT showing three-dimensional (3D) representation of ultrastructure within the chloroplast of *B.pilosa*. **(A)** Reconstructed image of the 0°-projection SXT acquired from TPS24A electron as 2D micrograph (slice 125/250) showing lipid bodies (red arrowhead), thylakoid (cyan arrowhead) and starch granules (orange arrowhead) according to the X-ray absorbance level. **(B)** 3D representation of lipid bodies (blue), chloroplasts (green), thylakoid system (cyan) and starch granules (brown) overlaid on the reconstructed electron micrograph (slice 125/250). **(C)** 3D representation of the reconstructed tomography tilted at -49° off the x-axis and overlaid on slice 125/250, showing starch granules (brown) and thylakoid system (cyan). **(D)** 3D representation of lipid bodies (blue), chloroplasts (green), thylakoid system (cyan), and starch granule (brown). **(E)** 3D representation of one chloroplast (green) containing thylakoid system (cyan) and starch granules (brown) tilted at -49° off the x-axis, showing the outer membrane (green), starch granule (orange) and thylakoid system (cyan). **(F)** 3D representation of the thylakoid system (cyan) and starch granules (brown) tilted at -49° off the x-axis. All scale bar in **Figure 4**= 1 µm.

In contrast to *A. thaliana*, *B. pilosa* chloroplasts exhibited a significantly more robust ultrastructure. Tomographic reconstructions revealed broader thylakoid lamellae forming dense grana stacks and larger starch granules, both in size and number (**Figures 4A, B**). Also, the presence of a large near spherical object was noted within each chloroplast of *B. pilosa* (**Figure 4A**). Furthermore, several electron-dense, spherical structures were observed in the cytosol near chloroplast peripheries (**Figure 4C**), which were rendered in 3D as lipid bodies (blue; **Figure 4D**; **Supplementary Video S3**).

When compared in 3D models, *B. pilosa* chloroplasts appeared thicker and more layered than those of *A. thaliana*, with starch granules (orange) occupying larger proportions of stromal space and thylakoid membranes (cyan) forming highly stacked granae (**Figures 4E, F**; **Supplementary Video S4**). This provides compelling evidence that SXT can reveal interspecific differences in subcellular organization and plasticity, which are likely shaped by evolutionary and ecological adaptations.

## 4 Discussion

Although plant protoplast isolation protocols have advanced significantly over the past decade, most plant cell structural studies still rely heavily on transmission electron microscopy (TEM), scanning electron microscopy (SEM), and confocal microscopy (Zechmann, 2023; Okumura et al., 2018; Caldwell and Iyer-Pascuzzi, 2019). These tools remain indispensable due to their high resolution or real-time capabilities. However, they are limited by sample preparation requirements, such as sectioning and chemical fixation, and in the case of confocal microscopy, by restricted imaging depth and labeling constraints.

The average thickness of plant cells—particularly those of leaf mesophyll—poses a major obstacle for volumetric imaging techniques such as SXT, which typically penetrates up to ~15 µm in biological samples (Mathers et al., 2018). While recent efforts utilizing cryogenic electron tomography (cryo-ET) have allowed imaging of *A.thaliana’s* protoplasts (Sanchez Carrillo et al., 2023), the resulting tomograms are limited to depths of 100–200 nm, insufficient for visualizing entire organelles or their dynamic spatial organization in 3D.

The TPS-24A1 beamline has recently emerged as a powerful platform for high-resolution, label-free 3D imaging of biological samples, including mammalian cells such as cancer cells (Huang et al., 2024; Cheng et al., 2025; Lai et al., 2023). However, its application in plant biology has been largely underutilized, primarily due to the intrinsic thickness of plant tissues and the associated challenges in beam penetration. By successfully adapting the protoplast isolation procedure for compatibility with SXT, we were able to overcome this barrier and directly image plant protoplasts within the critical penetration range (~5–15 µm) required for high-contrast volumetric reconstructions (**Figure 1**; **Figures 2A, B, D, E**).

The imaging success at TPS-24A1 is enabled by its use of photon energy within the “water window” (284–543 eV), which allows strong contrast between carbon-rich structures and water-based cytoplasmic regions (Stasio et al., 2000). In this range, denser hydrocarbon-based molecules such as lipids and starch granules absorb more X-rays and thus appear as darker features in the tomography, while water-rich regions appear lighter. Based on this principle, we interpreted the dark gray areas in our tomograms as starch granules and the black, highly absorbent features as lipid bodies or long-chain carbon compounds (**Figures 2C, F**).

Further analysis of the reconstructed SXT images of *A. thaliana* protoplasts allowed for the identification of multiple chloroplasts along with their internal ultrastructures (**Figure 3**). Using IMOD software, we were able to differentiate suborganellar components based on grayscale contrast, with thylakoid membranes and starch granules clearly distinguishable (blue and orange arrows, respectively; **Figures 3A, B**). The reconstructed tomograms were then rendered into 3D representations, illustrating the organization of thylakoid membranes and starch granules within the chloroplasts (**Figure 3C**). These SXT-based reconstructions revealed a more open and robust internal architecture compared to conventional transmission electron microscopy (TEM) images, where chloroplasts are often depicted as compact oblate spheroids with densely packed thylakoid stacks (Zechmann, 2023). Notably, the 3D reconstructions of *A. thaliana* chloroplasts obtained via SXT showed that starch granules are clustered along the stroma and lamellae near the chloroplast envelope, and the thylakoid network appeared more spatially distributed (**Figures 3D–F**, **Supplementary Video S2**). These differences likely stem from the advantages of cryogenic preservation in SXT, which avoids artifacts associated with chemical fixation and dehydration often used in TEM (Carrera et al., 2021; Giełwanowska et al., 2015, Yongzong Lu et al., 2019, Zechmann, 2023).

To demonstrate the broader applicability of SXT in studying protoplasts derived from non-model plant species, we applied our protocol to *B. pilosa*, a member of the Asteraceae family known for its high levels of oxylipins and other oxygenated fatty acid derivative (Scott et al., 2022; Chen et al., 2020). These compounds are of particular interest due to their roles in plant stress response and medicinal value. SXT imaging of *B. pilosa* protoplasts revealed numerous dark, lipid-dense structures dispersed throughout the cytosol, which we interpreted as lipid bodies (**Figures 4A, B**). This observation aligns with the known metabolic profile of *B. pilosa*, which accumulates long-chain fatty acids and triglycerides in both vegetative and reproductive tissues.

Interestingly, within all chloroplasts of *B. pilosa*, we observed a distinct, large, spherical suborganellar structure—approximately 3 µm in diameter—positioned near the center of each chloroplast (**Figures 4B, C**). While previous reports have identified smaller vesicles in chloroplasts of *A. thaliana* and *Lactuca sativa*, associated with lipid trafficking or plastid–cytosol communication (Gurkan et al., 2006; Karim et al., 2014), the size and consistency of this vesicular structure in *B. pilosa* appear unique. Notably, some of these vesicles appeared to harbor starch granules (**Figures 4B, D, E**), suggesting a potential role in carbohydrate storage or inter-organelle exchange. However, the biological significance of this structure remains uncertain. Further studies employing correlative imaging and molecular markers will be essential to clarify its origin and function. It is worth noting that *B. pilosa* is highly tolerant to drought and high temperature, and the ability to store large quantities of starch or lipids in its mesophyll cells may contribute to its adaptive success. Whether these large vesicles are a conserved feature among Asteraceae or specific to *B. pilosa* remains an open question for future investigation.

## 5 Conclusion

In summary, synchrotron radiation-based SXT enables high-resolution, three-dimensional imaging of plant cells in their near-native, fully hydrated state without the need for physical sectioning, chemical fixation, or staining. Our findings demonstrate that SXT provides an unprecedented view into the ultrastructure of plant protoplasts along the z-axis, revealing organelle morphology and spatial organization that are often altered or masked by traditional electron microscopy methods. While TEM continues to provide higher resolution for thin sections, it lacks volumetric information and may distort subcellular architecture due to sample dehydration and embedding. In contrast, SXT preserves cellular integrity and captures volumetric ultrastructure across the whole protoplast, providing insights into features such as chloroplast stroma, thylakoid networks, and starch granule distribution with superior three-dimensional fidelity.

An unexpected observation was the presence of a large, vesicle-like structure within *B. pilosa* chloroplasts—an ultrastructural feature not previously reported in model species. The consistent presence of these structures, alongside abundant lipid bodies, underscores SXT’s capability to identify species-specific subcellular features that may be involved in stress adaptation or specialized metabolic processes. These findings highlight the untapped potential of SXT in expanding our understanding of structural diversity across non-model plants.

Beyond structural imaging, the SXT platform offers advanced capabilities in spectral imaging by tuning the photon energy near the absorption edges of biologically relevant elements. Of particular interest are transition metals such as iron (Fe), cobalt (Co), and nickel (Ni), which play essential roles in plant physiology, including photosynthesis, nitrogen fixation, and reactive oxygen species (ROS) regulation. By adjusting the beam to energies near their respective L-edges or K-edges, future studies may enable *in situ* localization and quantification of these metal ions at nanometer-scale resolution within plant cells. This opens a new frontier in plant cell biology: the potential to map elemental composition and trace metal dynamics alongside structural context—without the need for external stains or labels.

As SXT technology continues to advance, especially in spectral imaging and tomographic reconstruction algorithms, we anticipate that it will become an indispensable tool for plant biologists seeking to unravel the interplay between structure, metabolism, and environment. This approach could be particularly transformative for studying root–soil interfaces, micronutrient uptake, and biotic/abiotic stress responses in both model and non-model plant systems.

## Data Availability

The original contributions presented in the study are included in the article/**Supplementary Material**. Further inquiries can be directed to the corresponding author.
